# Spirituality, symptom burden and palliative care needs assessed by the Palliative Outcome Scale in those with cancer: a cross-sectional study

**DOI:** 10.3332/ecancer.2026.2089

**Published:** 2026-03-12

**Authors:** Gustavo Santos Paiva Laender Moura, Laura Gonzaga de Carvalho Bonifácio, Fernando Pedreschi Bernardes, Livia Regina Gonçalves e Silva, Vivian Marques Miguel Suen, Maria Izaura Sedoguti Scudeler Agnollitto, Rosana Aparecida Spadoti Dantas, Nereida Kilza da Costa Lima, Mevhibe Banu Hocaoglu

**Affiliations:** 1 Department of Internal Medicine, Ribeirão Preto Medical School, University of São Paulo, Ribeirão Preto, SP 14049-900, Brazil; 2 Base Hospital of the Federal District, Federal, Brasilia 70330-150, Brazil; 3 University of Ribeirão Preto, Ribeirão Preto, SP 14096-900, Brazil; 4 University Center of the Central Plateau Apparecido dos Santos, Federal, Brasilia 72445-020, Brazil; 5 Ribeirão Preto College of Nursing, University of São Paulo, Ribeirão Preto, SP 05508, Brazil; 6 Cicely Saunders Institute of Palliative Care, Rehabilitation and Policy, King's College London, WC2R 2LS London, UK; a https://orcid.org/0000-0003-1298-8557; b https://orcid.org/0000-0001-6155-5120; c https://orcid.org/0009-0003-0724-9714; d https://orcid.org/0009-0000-6779-304X; e https://orcid.org/0000-0001-6165-5746; f https://orcid.org/0000-0003-0091-4307; g https://orcid.org/0000-0002-3050-7000; h https://orcid.org/0000-0002-7139-8883; i https://orcid.org/0000-0003-1417-7117

**Keywords:** spirituality, symptom burden, palliative care, cancer, cross-sectional study

## Abstract

**Background:**

Understanding symptom prevalence and palliative care needs is essential to advancing palliative care research in South America. This study aimed to assess these outcomes using the Palliative Outcome Scale (POS) and explore their relationship with the self-rated importance of spirituality.

**Methods:**

A cross-sectional study was conducted among adult persons with cancer receiving palliative care at an outpatient clinic in a Brazilian public hospital. Data were collected through structured interviews using the POS, which assesses physical and psychosocial symptoms, communication, practical concerns and information received. Spirituality was measured using a self-rated single-item question. Associations between symptom burden and spirituality were analysed using *t*-tests and logistic regression was used to examine the relationship between spirituality importance and inadequate pain control, adjusting for potential confounders. Statistical significance was set at *p* < 0.05.

**Results:**

Sixty-seven patients (mean age 60 ± 13.8 years; 68.6% female) participated. Pain (77.6%), nausea (27%), weakness/fatigue (20.9%) and psychological distress (19.4%) were the most prevalent symptoms. Most had metastatic cancer (62.7%), and 32.8% were undergoing chemotherapy. Patients rating spirituality as highly important had significantly lower pain scores (mean 1.4 ± 1.4 versus 2.6 ± 0.9, *p* = 0.006) and were over ten times less likely to report inadequate pain control (odds ratios = 10.74, 95% confidence intervals: 1.87–203.82, *p* = 0.028).

**Conclusion:**

Our findings underscore the high prevalence of physical, psychological and social challenges among persons with cancer receiving palliative care. Symptom patterns aligned with global data, suggesting a universal burden. The strong association between spirituality and pain highlights the protective role of spiritual well-being in mitigating suffering, reinforcing the need to integrate spiritual care into palliative frameworks. These results offer valuable insights for guiding clinical practice, shaping international policy and informing future research to optimise care delivery and patient outcomes.

## Background

Across much of South America, including Brazil, palliative care services remain insufficient despite being recognised as essential to universal health coverage. Access is hindered by structural inequalities, limited integration into health systems and an undertrained workforce [1]. According to Clark *et al* [2], Brazil is at an intermediate stage of palliative care development, marked by regional disparities and inadequate access to essential medications such as opioids. These gaps result in many patients – particularly those with advanced cancer – experiencing unmanaged symptoms and avoidable suffering. Expanding research capacity and generating local data have been identified as key strategies to guide the development of equitable and effective palliative care in the region [1].

The routine assessment of symptom burden is central to timely, patient-centered care. In advanced cancer, symptoms are often severe, dynamic and multidimensional and early symptom monitoring and multidomain assessment are essential to guide care planning [3]. Similarly, the CovPall-Symptom study [4]. demonstrated that symptom-focused interventions – using instruments such as IPOS-COV – were associated with rapid improvements in pain, breathlessness, anxiety and agitation, even among patients with limited survival. Tools such as the Palliative Outcome Scale (POS) offer a validated means of capturing not only physical symptoms like pain and fatigue but also psychological and practical concerns [5].

Spirituality is a fundamental, though often overlooked, dimension of palliative care [6].. Rooted in the concept of total pain [7], spiritual distress can profoundly affect patients’ experiences of illness and dying. In South America, where religious and spiritual values are culturally prominent, spirituality frequently influences coping, meaning-making and perceptions of suffering [1, 8]. However, spiritual needs are rarely assessed systematically and are often neglected in clinical practice [9]. Research shows that unmet spiritual needs are associated with higher psychological distress and lower quality of life in palliative care settings [8, 10]. While spiritual care has the potential to alleviate suffering and foster dignity, it remains an underdeveloped area of care in Brazil and elsewhere in the region [1].

Therefore, this study aimed to measure the symptom prevalence and palliative care needs using the POS and to explore whether self-rated spirituality importance correlates with pain burden.

## Methods

### Study design and setting

This cross-sectional study was conducted at a palliative care outpatient clinic within a public hospital in the capital of Brazil. The hospital is the primary referral center for tertiary care in the Brazilian midwestern region. It offers specialised services for managing all cancer types, including consultations, diagnostic procedures and treatment modalities such as surgery, radiotherapy, clinical oncology and palliative care. The hospital-based palliative care team offers comprehensive inpatient and outpatient consultations. Currently, the hospital has 634 beds and employs over 4,000 individuals.

### Participants

Adult persons with cancer receiving palliative care who could complete an interviewer-administered questionnaire were eligible to participate. Exclusion criteria were patients attending their first appointment or currently hospitalised. All eligible patients attending the clinic were invited to participate. Participants were included and interviewed between 1st and 31st January 2021. Secondary data collection from hospital records continued until 12th January 2024. Data were collected using REDCap (Vanderbilt University, Nashville, TN, USA).

### Measures and procedures

The POS is a multidimensional questionnaire comprising ten Likert-scale questions that address essential aspects of palliative care. Responses range from 0 (best) to 4 (worst) based on descriptive answer options. Each item can be scored and considered individually or summed to yield a total score ranging from 0 to 40, with higher scores indicating higher palliative care needs. The scale addresses a range of issues, including physical symptoms such as pain, emotional concerns like anxiety and practical challenges. Additionally, item 11 allows patients to report their main problems in an open-text format [5, 11, 12]. The Brazilian-Portuguese version of the POS questionnaire (POS) [13]was used for data collection. Due to COVID-19 restrictions, patient interviews were conducted via voice call. After obtaining written consent, the researchers conducted the interview and read the POS items verbatim to patients or their relatives, who relayed them for direct responses.

After completing the POS, patients were asked: ‘On a scale of one to five, where one signifies little importance, and five signifies maximum importance, how important is spirituality in your life?’ This question was adapted from spiritual belief system, personal spirituality, integration, ritual restrictions, implications and terminal events since POS does not include spirituality-related items [14].

Sociodemographic data, including age, gender, marital status and distance from household to hospital, were collected from hospital records. Clinical data corresponding to the palliative care medical visit nearest to the interview were collected from patient charts. Clinical data included cancer site, staging, treatments, comorbidities, medications, patient-reported symptoms, functionality status assessed with the Brazilian Portuguese adaptation of the Palliative Performance Scale (PPS) [15]and medical management.

Patient-reported symptoms during the palliative care medical consultation were systematically categorised into body systems, including gastrointestinal, cardiorespiratory, neurological, psychiatric, musculoskeletal and genitourinary. The classification process involved careful consideration of symptom presentation and context. The primary author conducted the initial classification and ambiguous cases were reviewed by the author panel for consensus.

### Analysis

Continuous variables were summarised using means, standard deviation (SD), medians and interquartile ranges (IQRs). Categorical data were presented as frequencies. POS results are reported without grouping response items. Binary logistic regression analysis examined the relationship between the self-rated importance of spirituality and pain control. The outcome variable, pain control, was derived from POS item 1 and categorised as 0 for adequate pain control (responses = 0 or 1) and 1 for inadequate pain control (responses = 2–4). The predictor variable, self-rated importance of spirituality, was coded as 0 for maximum importance (response = 5) and 1 for little to moderate importance (responses = 1–4). A baseline null model, including only the intercept, was fitted to establish the initial deviance. Subsequently, a univariate logistic regression model was constructed to evaluate the effect of self-rated spirituality on the odds of experiencing inadequate pain control. The goodness-of-fit of the models was assessed using a chi-squared test for the reduction in deviance between the null model and the fitted model, with statistical significance set at a two-sided alpha level of 0.05. The results of the logistic regression model were expressed as odds ratios (ORs) with 95% confidence intervals (CIs). Model performance was further evaluated using deviance and the Akaike Information Criterion. All analyses were conducted using R version 4.4.1 with the glm() function from the base package. No adjustments or imputations were performed for missing values.

## Results

**Figure 1.**
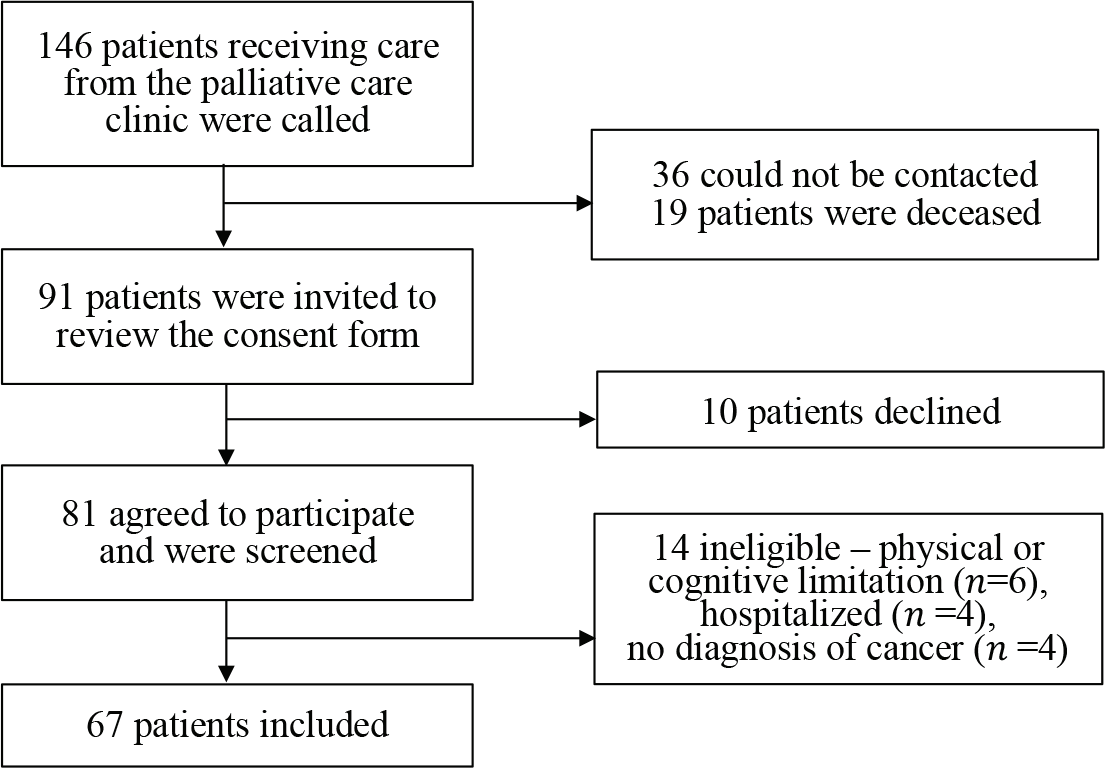
Inclusion diagram.

Sixty-seven patients completed the POS (Figure 1). The mean age was 60 ± 13.8 years; most were female (68.6%) and single (43.3%). The POS mean total score was 15.5 ± 6.97, and the median PPS was 70% (IQR 60–80). The most prevalent primary cancer sites were uterine cervical cancer (23.9%) and gastrointestinal (13.4%). Stage IV metastatic cancer represented 62.7% of cases, and 32.8% were currently undergoing chemotherapy. The median number of comorbidities was 3 (IQR 2–4), the most prevalent being hypertension (47.8%) and type-2 diabetes (19.4%). Malignancy-related comorbidities affected 49.3% of patients, with thrombosis being most common at 11.9%. Table 1 shows patient characteristics, and Table 2 reports individual POS item results. In responding to the POS questionnaire, 54.7% did so independently, while 42.2% had a family member or friend relay the questions to them for direct responses.

Fifty-six patients (83.6%) rated spirituality as being of maximum importance, with a mean pain burden score (POS item 1) of 1.4 ± 1.4. Conversely, 11 patients (16.4%) rated spirituality as having little to moderate importance, with a mean pain burden score of 2.6 ± 0.9. The model with spirituality importance significantly improved the fit compared to the null model (*χ*^2^_(1)_ = 7.886, *p* = 0.005). Patients who rated spirituality as having little to moderate importance had significantly higher odds of reporting worse pain burden compared to those who rated spirituality as being of maximum importance (OR = 10.74, 95% CI: 1.87 to 203.82, *p* = 0.028).

Pain was the most prevalent symptom (77.6%) (Table 3), and analgesics were the most frequent medications (82.1%) (Table 4), with a median of two per patient (IQR 1–2), opioids (65.7%) being the most common. The leading analgesic adjuvants were gabapentinoids (43.3%). Additionally, pain management was the most common medical intervention, with 34.3% receiving additional prescriptions or dosage adjustments for pain control. A history of opioid intolerance was reported for 16.4% of patients, primarily due to gastrointestinal symptoms.

Gastrointestinal symptoms affected 50.7% of patients, with nausea being most common at 27% (Table 3). Overall, 37% used antiemetics (Table 4), mainly 5-HT3 receptor antagonists (68.0%) and dopamine receptor antagonists (32.0%). Medical intervention for nausea control required medication changes in 10.4% of cases. Constipation was reported by 20.9% of patients, and 46.3% used laxatives. Additionally, constipation required medication adjustments in 16.4% of cases. The most common laxatives were bisacodyl (16.4%), lactulose (14.9%) and mineral oil (13.4%).

## Discussion

The POS scores reveal critical insights into the palliative care needs and symptom burden of patients, particularly in the domains of pain burden (POS item 1), anxiety (POS items 3 and 4), difficulties with information and communication (POS items 5 and 6), and time wasted on appointments (POS item 9), which showed median scores of 2 or higher.

These findings emphasise gaps in supportive care that may exacerbate distress and hinder quality of life. For example, the elevated scores for information and practical matters highlight the need for enhanced communication, education and practical support to address unmet needs. Focusing on these areas could significantly alleviate psychological burden and improve patient outcomes.

Notably, patients who rated spirituality as less important were over ten times more likely to report worse pain burden, suggesting that spirituality may serve as a protective factor against pain perception or as a mediator for coping mechanisms. Our findings align with previous research demonstrating that higher spirituality scores are associated with improved pain tolerance and reduced psychological distress, as shown in Koenig's [16] comprehensive review, highlighting spirituality's protective role in physical and mental health. These findings are consistent with evidence suggesting that spirituality and religious coping mechanisms often play crucial roles in fostering resilience in patients facing chronic or severe illnesses [17]. Importantly, studies such as Pargament's longitudinal analysis reveal that positive religious coping (e.g., seeking spiritual support) is linked to lower pain and distress levels, while negative coping mechanisms correlate with worse health outcomes [17].

The convergence of these findings with Dame Cicely Saunders' ‘total pain’ concept further reinforces the need for holistic care approaches. ‘Total pain’ highlights the interconnectedness of physical, psychological, social and spiritual dimensions of suffering [18]. Saunders' foundational work in palliative care emphasises that untreated spiritual distress can exacerbate other dimensions of pain, reinforcing the necessity of holistic care. Healthcare providers can mitigate these interdependent factors by addressing spiritual needs, potentially improving patients' overall well-being. These findings reinforce the growing consensus in the literature that spiritual care is a vital component of comprehensive palliative care and quality of life [19]. For example, Balboni *et al* [19] demonstrated that persons with cancer receiving spiritual care report better quality of life and are more likely to receive hospice care near the end of life.

Our findings on the top ten most prevalent symptoms are consistent with a comprehensive review of 35 studies from 15 countries on palliative care for people with cancer. Except for dyspnea, which we observed less frequently (6% versus 21%), the top ten symptoms remain constant [20]. Identifying a consistent pattern of symptom burden and palliative care needs across diverse populations underscores the universality of these challenges and their relevance to global health.

From an international policy perspective, these findings highlight the urgent need to prioritise symptom management in palliative care frameworks. Standardised guidelines could address common symptoms consistently across settings, ensuring equitable care delivery. Additionally, the observed global consistency in symptom burden provides solid evidence for directing resources toward training healthcare providers, integrating multidisciplinary care teams and expanding access to palliative care services in underserved regions [21, 22]. In such regions, limited access to trained providers and essential medications exacerbates disparities in care delivery, further emphasising the importance of resource allocation.

These findings also emphasise the importance of aligning research priorities with the most burdensome symptoms identified in palliative care populations. Future international collaborations could focus on evaluating interventions that address common and localised symptom needs, tailoring solutions to regional contexts while maintaining global relevance. Ultimately, a deeper understanding of symptom burden patterns across diverse populations can inform policies that promote universal access to high-quality palliative care, improving the quality of life for patients worldwide [23].

The high prevalence of psychiatric symptoms observed in this study, reflected in anxiety-related POS items, further emphasises the importance of integrating psychosocial and spiritual care into treatment plans. Previous studies have demonstrated that addressing spiritual distress can reduce anxiety and depressive symptoms while improving patients' sense of meaning and purpose [24, 25]. For instance, Lucchetti *et al* [24] highlighted how spiritual care integration into general practice supports patients in coping with existential challenges. Similarly, Koenig's [25] review illustrates its role in reducing depressive symptoms and enhancing psychological resilience. The strong association between spirituality and pain burden identified in our study underscores the necessity of attending to spiritual well-being as part of a holistic, multidisciplinary approach to care.

In addition to spiritual care, targeted interventions addressing the interplay of physical, emotional and spiritual factors could help reduce the burden of suffering in patients with chronic pain or life-limiting illnesses. Approaches such as mindfulness-based stress reduction (MBSR) [26], meaning-centered therapy [27–29]and chaplaincy services have shown promise in enhancing resilience and fostering adaptive coping mechanisms [30]. For example, a recent randomised trial by Hoge *et al* [26] found that MBSR was as effective as escitalopram in reducing anxiety symptoms, offering a non-pharmacological option for patients. Similarly, meaning-centered therapies like those studied by Breitbart *et al* [29] and Kissane *et al* [28] have significantly improved psychological well-being and reduced existential distress among advanced persons with cancer. As highlighted by Balboni *et al* [30] Chaplaincy services were associated with improved quality of life and greater emotional support for patients with advanced illnesses.

Incorporating spiritual care into clinical practice could involve routine screening for spiritual well-being, providing access to chaplaincy services or training healthcare providers to identify and address spiritual needs. Furthermore, our findings align with the growing recognition of spirituality as a dimension that interacts with psychological and social support to influence health outcomes [31]. George *et al* [31], for example, emphasised the multifaceted role of spirituality in enhancing social support networks and mitigating the effects of stress. These findings highlight the importance of further research into the mechanisms underlying these relationships. For instance, future studies could explore whether spirituality exerts its effects through direct modulation of pain perception, enhancement of psychological resilience or indirect pathways involving social or community support. Longitudinal studies are particularly needed to clarify causal relationships between spirituality and pain burden and to evaluate the long-term effectiveness of spiritual interventions, which could refine holistic care models and optimise outcomes for vulnerable populations.

### Implications for clinical practice

Although many persons with cancer are followed outside specialist palliative care teams, our findings offer practical recommendations to strengthen the capacity of general clinicians who provide supportive and palliative care. First, routine use of brief, validated tools such as the POS can facilitate early identification of uncontrolled symptoms, psychosocial distress and communication gaps, allowing timely and targeted interventions even in non-specialist settings. Second, given the strong association between spirituality and pain burden observed in this study, clinicians should incorporate simple spiritual screening questions into routine assessments and, when appropriate, collaborate with chaplaincy or community spiritual support services. Third, the high prevalence of anxiety, information needs and practical concerns underscores the importance of consistent communication, shared decision-making and clear explanations about prognosis, treatment goals and care plans. Finally, strengthening access to basic palliative care training – focused on pain management, opioid titration, psychological support and spiritual care – can help non-specialist clinicians deliver more holistic, person-centered care, particularly in regions with limited palliative care workforce. These strategies may improve symptom control, enhance quality of life and reduce avoidable suffering among patients with advanced cancer who do not have access to specialist palliative teams.

### Limitations

Several limitations should be considered when interpreting the findings of this study. First, the measure of spirituality was derived from a single self-reported question adapted for this study, as the original POS tool does not include spirituality-related items. While this approach provided valuable insights, it may not fully capture the multifaceted nature of spirituality, limiting its precision as a predictor. Second, categorising pain control into binary outcomes (adequate versus inadequate) based on POS item 1 responses may have oversimplified a complex variable. Future studies could adopt more detailed categorisations or continuous modeling to better reflect the spectrum of pain control experiences.

Third, the cross-sectional design of this study limits causal inferences. While a significant association between self-rated spirituality and pain control was observed, the directionality and mechanisms of this relationship remain unclear. Nevertheless, the study's primary aim – to assess symptom prevalence and palliative care needs among persons with cancer in Brazil – supports the appropriateness of a cross-sectional approach. Furthermore, our findings' alignment with the existing literature and theoretical frameworks reinforces the validity of the observed associations. Future research employing longitudinal or experimental designs could better address these gaps and clarify causal pathways.

Fourth, the study was conducted in a single public tertiary hospital in Brazil's capital, which may limit the applicability of the results to other settings or populations. However, the consistency of our findings with a comprehensive review spanning 15 countries supports their broader relevance and validity. Replicating this research in more heterogeneous populations is recommended to confirm these results.

Finally, the reliance on patient-reported symptoms and medical records introduces potential recall bias and data inaccuracies. Despite this limitation, this approach offered valuable insights into symptoms and palliative care needs that might otherwise be overlooked.

## Conclusion

This study highlights critical gaps in palliative care, emphasising the interconnectedness of physical, psychological, social and spiritual dimensions of suffering. Our findings demonstrate the significant role of spirituality as a protective factor against pain burden, with patients who rated spirituality as less important being over ten times more likely to report inadequate pain control. The elevated POS scores in domains such as pain, anxiety and communication further underscore the need for holistic approaches that address both physical symptoms and psychosocial support.

From a global health perspective, the consistency of symptom burden patterns across diverse populations reinforces the urgency of prioritising symptom management in palliative care frameworks. Standardised guidelines expanded access to multidisciplinary care teams, and targeted training for healthcare providers are essential to address the unmet needs identified in this study. Integrating evidence-based interventions such as MBSR, meaning-centered therapy and chaplaincy services can further enhance resilience and foster adaptive coping mechanisms, particularly for patients with chronic pain or life-limiting illnesses.

## Conflicts of interest

The authors declare no conflicts of interest regarding the research, authorship and article publication.

## Funding

This research received no specific grant from any funding agency in the public, commercial or not-for-profit sectors. MBH is supported by the NIHR ARC SL. The views expressed in this article are those of the authors and not necessarily those of the NIHR or the Department of Health and Social Care.

## Ethical standards

The study adhered to the 1964 Helsinki Declaration and the Brazilian National Council of Health's Resolution 466/2012 for research involving human participants. Approval was obtained from The Strategic Health Management Institute of the Federal District's Ethical Review Board (CAAE: 38432120.1.0000.8153) on 10/30/2020, and all participants provided verbal informed consent before inclusion. The information presented does not include any details that could reveal the identities of the study participants. The findings are reported following STROBE [32].

## Data availability statement

The data supporting the study findings are available from the corresponding author upon reasonable request.

## Figures and Tables

**Table 1.** Patient demographic and clinical characteristics.

Patient characteristics (*n* = 67)	Count	% total
Gender (Female)	46	68.7
Current relationship status		
Single	29	43.3
Married or living with a partner	25	37.3
Widowed	7	10.4
Divorced	6	9
Distance to healthcare service		
15–30 km	27	40.3
30–45 km	22	32.8
>45 km	11	16.4
0–15 km	7	10.4
Primary cancer site (*n* = 67)		
Uterine cervical cancer	16	23.9
Digestive system	9	13.4
Breast cancer	8	11.9
Male reproductive	7	10.4
Head and neck	7	10.4
Other	6	9
Lungs	5	7.5
Hematopoietic and lymphoid	5	7.5
Other female reproductive System	4	6
Stage		
Metastatic (IV)	42	62.7
Locally advanced (III)	13	19.4
Limited (I and II)	12	17.9
Current chemotherapy	22	32.8
Previous cancer treatment		
Chemotherapy	56	83.6
Radiotherapy	43	64.2
Surgery	29	43.3
PPS (median, IQR	70%	(60–80)
Number of medications (mean, SD)	6.9	± 3.3
Comorbidities (*n* = 67)		
Number of comorbidities (median, IQR)	3	(2–4)
Primary	65	97
Hypertension	32	47.8
Type 2 diabetes	13	19.4
Secondary to the malignancy	33	49.3
Thrombosis	8	11.9
Pathologic bone fracture other (Pleural effusion)	5	7.5
Hydronephrosis, and actinic damage	9	13.5

PPS: Palliative Performance Scale

**Table 2.** POS scores (*n* = 67) from 0 (best) to 4 (worst).

POS items	Median (IQR)	Frequency (%) of item scores				
		0 (best)	1	2	3	4 (worst)
1 Pain burden	2 (3–0)	32.8	11.9	26.9	17.9	10.4
2 Other symptoms	0 (1–0)	56.7	19.4	16.4	6.0	1.5
3 Anxious/worried	2 (3–0)	37.3	6.0	22.4	22.4	11.9
4 Family anxious	2 (3–0)	26.9	10.4	17.9	28.4	16.4
5 Information	3 (4–1)	22.4	7.5	10.4	14.9	44.8
6 Share feelings	1 (3–0)	41.8	16.4	13.4	7.5	20.9
7 Life worthwhile	0 (1–0)	68.7	13.4	10.4	6.0	1.5
8 Feel good	1 (2–0)	38.8	16.4	19.4	16.4	9.0
9 Time wasted	2 (4–0)	43.3	–	25.4	–	31.3
10 Practical matters	1 (4–0)	50.0	–	12.1	–	37.9

For IQR, Q3 and Q1 are presented respectively

**Table 3.** Prevalence of patient-reported symptoms.

Signs and symptoms (*n* = 67)	Counts	% total
Pain	52	77.6
Gastrointestinal symptoms	34	50.7
Nausea or vomiting	18	26.9
Neurological	18	26.9
Weakness/fatigue	14	20.9
Constipation	14	20.9
Edema	14	20.9
Psychological distress	13	19.4
Disabling pain	12	17.9
Insomnia	12	17.9
Unintentional weight loss	11	16.4
Reduced appetite	9	13.4
Musculoskeletal	8	11.9
Genitourinary	7	10.4

**Table 4.** Prevalence of medications in regular use.

Medications (*n* = 67)	Counts	% total
Antiepileptic drugs	33	49.3
Laxatives	31	46.3
Antiemetic	25	37.3
Antidepressants	25	37.3
Proton pump inhibitor	23	34.3
Vitamins and supplements	16	23.9
Corticoids	9	13.4
Antipsychotics (Quetiapine, haloperidol)	6	9
